# Contribution to the Pathophysiology and Treatment of Varicoceles

**DOI:** 10.5334/jbr-btr.1453

**Published:** 2018-02-09

**Authors:** Peter Vanlangenhove

**Affiliations:** 1 University Hospital Ghent, BE

**Keywords:** Varicocele, Embolization, Glue

## Abstract

Anatomical differences between adults and adolescents and between left and right varicoceles were shown in this work. We designed a standardized and reproducible method for pressure measurement in the inguinal internal spermatic vein (ISV). We demonstrated that the mean absolute pressure in the ISV in the upright position is higher than the veno-capillary pressure in the testicle, and hence could impair spermatogenesis prompting the need for treatment in varicoceles.

Histoacryl transparent and Glubran2, the current commercially available adhesives for the treatment of varicoceles, do not differ with regard to efficiency, safety and tolerance during and after embolization. Both adhesives cause a mild pain in 30% of the patients in the week after embolization. The radiation exposure is low during embolizations of varicoceles with highly viscous liquid products. Therefore, the endovascular treatment with glue is an efficient, safe and tolerable method of treatment for varicoceles when applicable.

## Introduction

This thesis aimed at contributing to the pathophysiology and treatment of varicoceles. The research project encompasses five prospective studies and one retrospective study. The specific objectives of these clinical studies were to assess: (i) the safety, efficacy and tolerance of two types of glue and a non-gluing liquid embolic agent, in the endovascular treatment of varicoceles, (ii) to assess radiation burden for patient during varicocele embolization, (iii) to develop a standardized method to read the phlebographic anatomy of varicoceles and to test a different ontogenesis of varicoceles in adolescents and adults, and (iv) lastly to develop a standardized method to measure the intravascular pressure in the internal spermatic vein and to determine whether infertility in varicoceles can be explained by the principle of elevated hydrostatic pressure.

### Presentation, incidence and epidemiology of varicoceles

The vast majority of adolescents with varicoceles are asymptomatic [1]. It may be an incidental finding, being discovered at a routine school medical examination.

An adult patient with a varicocele complains either for scrotal pain or heaviness at the scrotal sac or for infertility problems. Approximately 15% of all men in the general population have a clinical varicocele. Between 19–41% of men investigated for infertility have a varicocele [2]. The incidence of varicocele in men with secondary infertility rises to approximately 70% [3]. This observation suggests that with age, the impact of varicoceles on fertility becomes paramount. Because varicosis prevalence increases with advanced age, Canales, et al. hypothesized and evidenced that the incidence of varicoceles in the elderly population would be greater [4].

The prevalence of varicoceles increased significantly at 13-years of age, somewhere at the start of puberty. Varicoceles are rarely found in boys six to nine years of age [5, 6, 7]. The incidence of varicocele reaches its maximum of 16% in boys after puberty [7]. Akbay, et al. detected a significant prevalence increase with age and an increasing testicular atrophy with puberty. He concluded that varicocele is a progressive disease.

Most studies based on venography and with large number of patients report a bilateral varicocele in 25% [8, 9, 10]. In studies using different diagnostic tools, bilateral varicocele was reported in a range between 40% and 60% [11, 12]. Gat, et al. questioned the fact that varicocele should be considered as a mainly left-sided disease. In reports on adolescents and infertile males, they detected a bilateral varicocele in more than 80% with venography [13].

The heredity of varicoceles and the potential transmission to first-degree relatives has been little investigated. Clinical varicoceles are more prevalent among first-degree relatives of patients with known varicoceles [14].

### Etiology, pathophysiology, diagnosis and treatment of varicoceles

The etiology and pathogenesis of varicoceles cannot be explained by one single theory. Valve dysfunction, ontogenetic collateral formation and the nutcracker phenomenon seem to act synergistically. Hyperthermia, elevated hydrostatic pressure and antisperm agents are suggested as possible causes for the pathophysiology how varicoceles induce infertility. However, the combination of patient’s lifestyle, genetic factors and the consequences of reflux into the pampiniform plexus (PP) are believed to contribute to the infertility.

Although venography stays the gold standard, the combination of physical examination, colour Doppler ultrasound and thermography has the highest sensitivity and specificity to diagnose a varicocele. Regarding infertility, criteria or grading, to decide which patients with a varicocele may or may not have benefit from treatment remain to be established.

Treatment of varicoceles can be performed by different open, mostly surgical techniques and by percutaneous, mostly endovascular techniques. Treatment of varicoceles for infertility or to prevent infertility remains controversial, because the majority of men with varicoceles are still fertile. At the moment, inguinal or subinguinal microscopic surgery gave the highest pregnancy rates, the lowest recurrence and lowest complication rates. But retrograde superselective glue embolization or sclerosing of the ISV are the best percutaneous alternative and can be performed on an outpatient basis under local anesthesia and with faster return to normal activities than surgery.

### Percutaneous endovascular occlusion

As the different surgical techniques for the treatment of varicoceles were developed in the 70–80 ties, endovascular embolization emerged as a valuable alternative.

Depending on the used embolic agent the occlusion can be restricted to the main ISV or extended to the collateral venous network. The first technique is the endovascular equivalent of the surgical ISV clipping, the latter has a similar effect than an extended microsurgical dissection and clipping of all veins. The first reports of retrograde embolization of the ISV are from the late 70ties [15, 16]. With a transfemoral Seldinger technique, a catheter is inserted in the RV for diagnostic phlebography and thereafter deep into the ISV for embolization. Embolic agents can be directly delivered through the diagnostic catheter or in a coaxial way through a microcatheter. The procedure is performed on an outpatient basis under local anesthesia on a tilted X-ray table [17, 18].

There are liquid (sclerosing agents and glue) and non-liquid (detachable balloons, gelatin sponges and coils) agents. Many investigators use a combination of these agents to treat varicoceles. Newer products like Onyx®, vascular plugs or detachable coils are used in case reports or in unpublished series [19, 20]. Liquid agents have the advantage that they can penetrate into the collateral venous network around the ISV.

### Liquid embolics

Sclerosing agents (sodium tetradecyl sulfate 3% (STS), sodium morrhuate, dextrose and aethoxysklerol (1–2 or 3%)) are the most commonly used liquid agents in Europe (not authorized in the USA for spermatic veins) [21]. During Valsalva maneuver, the sclerosing agent is injected as a pure liquid or mixed with air as foam at the level of the inguinal ring. Manual compression of the external inguinal ring is mandatory to prevent the agent penetrating into the PP, hence avoiding inflammation and thrombosis.

**Figure 1:**
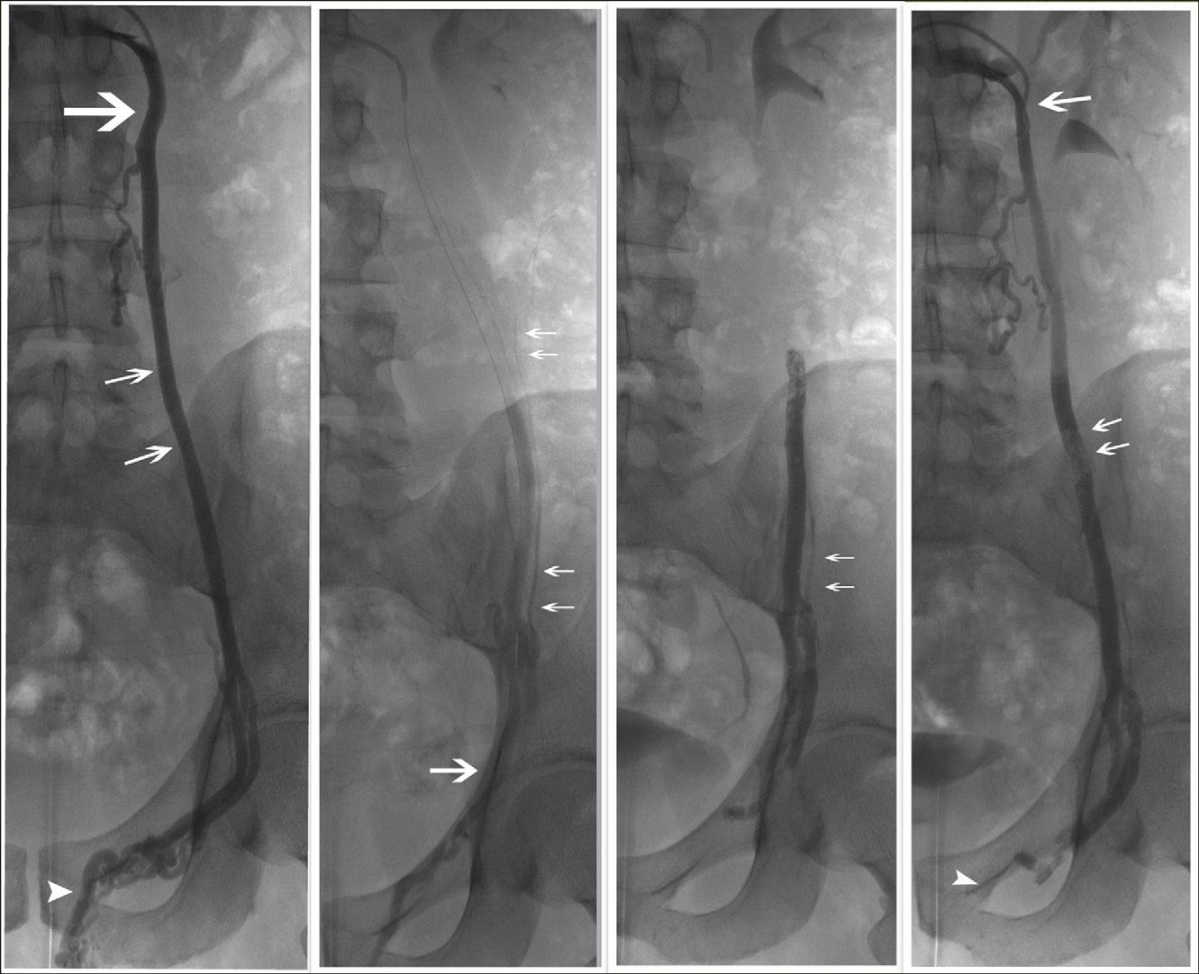
ISV embolization with glue. **Right**: Selective venography in erect position with the diagnostic catheter (arrow) in the outflow of the internal left spermatic vein. Internal spermatic vein insufficiency (small arrows) is proved with visualization of the pampiniform plexus (arrowhead). **Right middle**: Microcatheter (arrow) venography performed in horizontal supine position, revealed a small paraspermatic collateral (small arrows) originating from the lateral duplication of the inguinal internal spermatic vein. This anatomy forced us to reposition the microcatheter in the lateral bifurcation of the internal spermatic vein to be sure that the small collateral is also occluded during controlled injection of glue. Glue embolization will be started in the lateral branch at the level of the coxofemoral joint. During withdrawing of the microcatheter, the glue is pushed into the lateral branch, the small collateral and finally into the medial branch and the main internal spermatic vein then up to the level of the crista iliaca. **Left middle**: Embolization cast in the left spermatic vein with the glue located between the coxo-femoral joint and the crista iliaca including the small paraspermatic branch. **Left**: Control venography with the diagnostic catheter in the internal spermatic vein (arrow) demonstrates contrast agent up to the glue cast (small arrows) and the absence of contrast to the pampiniform plexus (arrowhead).

Tissue-adhesives (Cyanoacrylates) were first introduced by Kunnen, et al. in 1980 [22]. Glue is a less frequently used liquid agent which requires a coaxially catheter system for application (Figure 1) [10, 18, 23]. After verifying flow control with contrast agent injections during different table inclinations, the glue is sequentially injected through the microcatheter and pushed into the ISV as well as into the collaterals. As soon as the glue comes in contact with blood the polymerization process starts, and a permanent occlusion of the vessel occurs. The first glue used was a mixture of contrast agent and isobutyl-2-cyanoacrylate, IBCA (Bucrylate, Ethicon). Later on, IBCA was replaced by NBCA (n-Butyl-2-Cyanoacrylate or Embucrilate; Histoacryl transparent, Braun, Tuttlingen, Germany) because of possible carcinogenicity [24]. Today NBCA (Histoacryl) or NBCA-MS (Glubran2, General Enterprise Marketing, Viareggio, Lucca, Italy) is used with a mixture of Lipiodol Ultrafluid (Guerbert, France) which is necessary for the fluoroscopic visualization of the glue. Both, a higher percentage of Lipiodol and the prior injection of glucose 10% slow down the polymerization rate.

Onyx® (ev3, Irvine, California, USA) is a biocompatible ethylene-vinyl alcohol copolymer (EVOH) dissolved in dimethyl sulfoxide (DMSO) that in contact with blood solidifies as a kind of plastic. Onyx proved to be superior to tissue-adhesives for the occlusion and penetration of side-branches in cerebral embolization [25, 26]. Onyx embolization of varicoceles is feasible but still under investigation in our institution. Technical problems, combined with significant patient discomfort and a high radiation dose preclude, at the moment, its clinical use.

### Non-liquid embolics

Gelatin sponge is a water-insoluble, hemostatic sponge prepared from purified porcine skin gelatin. Gelfoam may be cut in small pieces that are injected through a diagnostic catheter and is able to absorb and hold many times its weight of blood and other fluids within its interstices. Gelatin sponge is a non-permanent occlusive agent, and therefore more used in combination with other permanent mechanical or liquid embolic agents [27].

Detachable balloons can be navigated through an introducer catheter and detached in the ISV. Depending on the size of the varicocele and the presence of collateral vessels, one or more 1- or 2-mm detachable balloons are used. Riedl and White were the first to treat varicoceles with detachable silicone balloons [28, 29]. After detachment of the balloon, a single radiograph was obtained, and follow-up abdominal radiographs were obtained three to four weeks after the embolization to exclude premature deflations. Balloons are frequently used in combination with other agents like coils or with *sandwiched* 70% dextrose (a sclerosing agent) [17].

Varicocele treatment with coils was introduced by Thelen, et al. who used the original 0.038-inch Gianturco coils [30]. Other fibered stainless-steel coils and platinum coils were developed, which could be delivered directly or in a coaxial technique. Coils can be pushable or, to improve correct placement, also detachable. Treatment includes occlusion of the mean ISV and major branches and all accessible collaterals starting from just above the inguinal canal up to the outflow of the ISV with the RV.

All percutaneous endovascular techniques have a low complication rate (<1%). Non-liquid agents (coils and balloons) and low viscosity liquids (sclerosing agents) have a high primary failure rate that ranges from 3–28% and increases when aberrant vessels are present [29, 31]. Recurrences occur in 2–19% [32, 33, 34, 35]. Most recurrences were related to a complex venous anatomy. Only embolization with high viscosity liquids (glue) has a reported low technical failure rate (<1%) and lower recurrence rates (< 2%) [10, 23, 36, 37]. The low technical failure rate is probably related to the use of a coaxial catheter system. A microcatheter with micro-guidewire facilitates to cross all competent valves. Better visualization and penetration of the embolic agent into collaterals could be responsible for the lower recurrence rate.

The need for contrast agent and the radiation exposure are the main disadvantages of the endovascular percutaneous technique. Severe allergic reactions to contrast agents are possible but very uncommon. Radiation exposure during embolization, with a subsequent 0.1% life-long risk for cancer, was reported in a retrospective study [38]. Radiation exposure during fluoroscopy is a concern but with radiation reduction techniques like lead shields and pulsed fluoroscopy, substantial reductions can be achieved.

The advantage of percutaneous embolization is that the patient can return to normal activities faster than with surgical treatment, since there is no incision and splitting of the abdominal muscles involved [39].

In our experience, we believe that retrograde liquid embolization under local anesthesia should be the treatment modality of choice for varicoceles. Solid embolic materials (metallic coils, detachable balloons) have the risk as in surgical clipping of leaving collaterals untreated, which could be responsible for recurrences. With low viscosity liquid sclerosing agents such branches and duplications can be occluded if injected in sufficient quantity and under Valsalva control. However, their low viscosity and visibility might increase nontarget embolization. Moreover, the sclerosing agent need time to obstruct the veins, so a waiting time of about 20 minutes is required to control its effect. A higher viscosity liquid like glue has the advantage of being radiopaque and of being a quick polymerization with definitive occlusion. The polymerization time can be reduced regarding the anatomical complexity of the ISV to allow the glue to penetrate into side branches [31, 35].

Percutaneous embolization with glue should be the first line of therapy in varicoceles. Superselective sclerotherapy is a good alternative in countries where tissue adhesives are not available.

### Embolization of varicoceles with cyanoacrylates

Since 1998, the FDA approved octyl-cyanoacrylates as a topical skin tissue adhesive. For butyl-cyanoacrylates this approval has been refused until 2007. As an endovascular adhesive and exclusive in cerebral interventions, only TRUFILL® n-Butyl Cyanoacrylate (n-BCA) Liquid Embolic System is FDA approved. GLUBRAN®2 is the only glue with a CE-mark for endovascular use.

Polymerization of glue in vessels produces an inflammatory reaction in the wall and in the surrounding tissues of the vessel, which changes in a chronic process resulting in fibrosis. Glue has to be mixed with iophendylate (IBCA), which makes the mixture radio-opaque during embolization. Beside its radio-opacity, iophendylate could change the polymerization time.

Kunnen introduced in 1980 the use of IBCA in varicocele treatment [22]. He reported a relative simple, safe and successful coaxially method to occlude the ISV in 35 patients on an outpatient basis. Since then, few additional series of varicoceles, embolized with glue were published (only nine publications) (Table 1). All reported a high success rate, low complication rate and low recurrence rate.

**Table 1:** Published series of varicoceles treated with glue.

	N (pt)	Tissue-adhesive	glue/ lipiodol	Technical failure	Technical complications	Clinical complications (mild to moderate discomfort)		Recurrences	Pregnancy rate

						during embolization	1 wk after embolization		

***Kunnen ’80***	35	IBCA	NA	0%	2.8% (1 glued catheter)			0% (PE+T+CDUS)	NA
***Comhaire ’85***	97	IBCA	NA	0%	NA			0% (PE+T+CDUS)	50.5%
**Mansfeld ’86**	30	NBCA	NA					3% (1pt on PE)	
**Nieschlag ’93**	33	NBCA	NA					6% (2pt)	33% (12 months)
**Heye ’06**	64	NBCA–MS (32) NBCA (32)	1/0.8 1/0.8	0%	17% (11 perforations)	3.28 NBCA–MS 3.23 NBCA***		2.1% (1pt)	
**Sze ’08**	9 8	NBCA NBCA+coils	1/3*	0%		5.9% (thromboplebitis		5.9%	
**Vanlangenhove ’12**	83	NBCA (54) NBCA–MS (58)	1/1.2 1/1	0%	1.2% (1 acute allergic reaction)	48% NBCA 38% NBCA–MS	57% NBCA 60% NBCA–MS	0	
**Pietura ’13**	17	NBCA	1/1	0%**	0%	100%	17.6%	0% (3 months/CDUS)	
Urbano ’14****	41	NBCA–MS	1/1	0%	0%	NA	17%	0% (12 months PE+CDUS)	

NA: Not available. *: Ethiodol. **: only phlebographic control in 3 of the 17 patients. ***: mean VAS pain score. ****: no coaxially catheter system/all patients took NSAID during 3 days. italic: same patient group. PE: physical examination, T: thermography, CDUS: colour Doppler ultrasound.

In 1985, Kunnen described a technical success of more than 99%, 0.46% complications (two glued catheters) and a 1.6% recurrence rate in a series of 435 patients [40]. In men with normal testicular volume, decreased follicle stimulated hormone below the average and a moderate impairment of the spermatogenesis, there was a pregnancy rate between 60% and 80%. Only Kunnen used IBCA until it was taken off the market in 1985. Since then, only studies of spermatic vein embolizations with only n-BCA or in combination were published. First, a German group from Berlin embolized 30 patients with NBCA mixed with Lipiodol with a technical success of 100% and a recurrence rate of 6% [41]. More recent reports showed a very high technical success (100%), very few complications (0–1.2%) and low recurrences (0–2.1%) [10, 23, 42, 43].

Two studies reported a higher recurrence rate of 6% [44, 45]. Although we expect some inflammatory reaction of the glue, thrombophlebitis of the PP is rarely reported [44]. We believe that a mild thrombophlebitis is present in most of the patients with a minimal self-limiting discomfort. Other patients have a mild to moderate pain reaction during the week after embolization localized in the abdominal flank, probably reflecting thrombophlebitis of the embolized ISV (not at the PP) [18].

Most of the investigators choose a 1/1 glue-lipiodol mixture to obtain a rapid polymerization and avoid penetration of the glue into the PP.

The largest population of varicoceles embolized with glue to date (3043 patients) is described by Kunnen in a book chapter. He reported a very low technical failure (<1%), a low complication rate (0.3%) and recurrence rate (2%) and a high pregnancy rate (50%) [36].

## Results

There are currently two commercially available tissue-adhesives that can be used for the embolization of varicoceles. The two adhesives differ only minimally in chemical formula, but polymerize in a different way. The more recently developed Glubran2, available since 2001, would have the same occlusion features as the older glue, hystoacryl transparent. The manufacture of Glubran2 obtained a CE label for intravascular use and alleged that Glubran2 has a higher stability and a lower toxicity.

We tested both glues (Histoacryl transparent, Glubran2) for differences in efficacy, safety and tolerance in a double-blind, prospective, randomized study. We found that both glues can be handled in the same way and that the embolic result is similar.

Technical success was 100%, this means that in all patients we could effectively block the reflux in the insufficient ISV. The procedure was very safe with no complications caused by the glue.

Concerning patients comfort we were the first to delineate a late inflammatory reaction to the cyanoacrylates. During the week after embolization, 59% of patients reported some discomfort, which was in 35% at least a bearable pain. There was no significant difference in discomfort between the two glues in the post-embolization week. Moreover, there was some mild acute inflammatory pain reaction during the embolization in 20% of the patients. There was no difference between both glue groups. Given these findings, patients should be informed that they might experience discomfort or mild pain during and after embolization of their varicocele. As this inflammatory reaction is self-limiting and mostly mild, we would not advise to prescribe NSAIDs prophylactically.

Because there is no difference between Histoacryl transparent and Glubran2, both glues can be used effectively and safely. However, it is medicolegally sensible to choose Glubran2 because it has a CE approval for endovascular use.

Another liquid embolic agent, which is non-adhesive, is ethylene-vinyl alcohol also called “Onyx”. Onyx is a plastic dissolved in dimethyl sulfoxide. After diffusion of the solvent, Onyx solidifies to a rigid cast, which can be pushed and extended in different directions. For these reasons, Onyx embolization has become the primary intravascular therapy for cerebral arterio-venous malformations (c-AVM) and dural fistulas (d-AVF). Because of the anatomical similarities between AVM and complex insufficient ISV, we thought that Onyx could be a potential embolic agent in complex varicoceles. Moreover, we know from extirpated AVM species that Onyx does not induce a post-embolization inflammatory reaction.

In a pilot study we tested Onyx as an embolic agent in ISV embolization. We found that Onyx was efficient and indeed better tolerated in the post-embolization period. However, there was an acute pain reaction during the injection of Onyx in nine of ten patients, prompting to stop the injection temporarily in six patients. The technique to inject Onyx as we do with glue, allowing only little reflux, demonstrated to be suboptimal and ensued a higher radiation dose. Further research should concentrate on adaptation of the embolization technique as used for c-AVM and on reducing the acute pain reaction by injection of intravenous anesthetics.

The first basic principle of radiation protection is *justification*. This means that the clinical benefit of the treatment has to outweigh the possible radiation-induced risks. The treatment of varicoceles to prevent infertility or to improve fertility, is very controversial. Other studies and our study, show that the gonadal dose in varicocele embolizations is definitely far below the lower threshold corresponding to the deterministic effect of temporary sterility (150 mSv).

Based on the in-vivo measured testes doses, we could estimate the risks of hereditary effects as being very low (mean value of 3.5 10^–6^). With respect to the risk of cancer mortality a mean value of 0.06% was calculated. The latter value is much lower than other typical interventional X-ray procedures. According to the NCRP publication 168, the obtained risks in our study population are requiring a *minor to moderate benefit* to justify the varicocele embolization procedure. The low dose and risk values obtained are probably linked to a set of specific radiation reducing measures. The latter optimization includes the use of pulsed fluoroscopy, the application of gonadal shielding and the careful collimation of the radiation beam.

Apart from the patient, the interventional radiologists are also at risk, especially those, which perform a high amount of procedures. Optimizing patient radiation exposure, will also reduce the patients scatter dose and hence the staff dose.

In everyday practice, varicoceles are treated because of symptomatology (pain and congestion of the scrotum) and the assumed association with infertility. In adolescents, varicoceles are mostly asymptomatic. Treatment of adolescent’s varicocele is advised to anticipate testicular hypotrophy and future fertility. To justify this prophylactic treatment, it is generally assumed that varicocele in children are an early stage of the varicocele in adults. Although the adult varicocele is usually symptomatic, treatment is requested in first place because of sperm dysfunction.

At a certain moment in my practice, I had the impression that the embolization of a varicocele in a child seemed a technically less challenging procedure than in an adult. I thought that *the easier procedure* was due to the absence of competent valves and a straighter anatomy in adolescents. These observations could point at a different phlebographic anatomy between adolescents and adults, and indirectly disclose a different pathophysiology of varicoceles.

To investigate this hypothesis, we set up a retrospective study to compare phlebographic radio- anatomical landmarks between adolescents and adults. For this purpose, we used the selective phlebographies that were performed prior to the embolization of the varicocele (Table 2a) (Figure 2a).

**Table 2a:** Phlebographic characteristics of the left insufficient ISV in adults and adolescents.

Phlebographic characteristics of the left ISV		Adolescents N = number adolescents/total N = 191(%)*	Adults N = number adults/total N = 218 (%)*	P-value

Spontaneous visualization of the ISV		164/190 (86.3)	154/214 (72.0)	0.001
Incompetence of the outflow valve**		128/190 (67.4)	125/216 (57.9)	0.052
Outflow of the ISV in the RV	Single outflow	179/191 (93.7)	189/218 (86.7)	0.021
	Complex outflow	12/191 (6.3)	29/218 (13.3)	
Reno-spermatic bypass	Absent	142/191 (74.3)	175/218 (80.3)	0.157
	Complete	49/191 (25.7)	43/218 (19.7)	
Mean nr. of competent valves below the outflow valve		0.17	0.33	0.000
Duplication of the ISV	Solitary ISV	130/191 (68.1)	127/218 (58.3)	0.052
	Multiple/duplication	61/191 (31.9)	91/218 (41.6)	
Paraspermatic veins		178/191 (93.2)	204/218 (93.6)	1.000
Collaterals	collaterals	108/191 (56.5)	154/218 (70.6)	0.030
	lateral collaterals	92/191 (48.2)	128/218 (58.7)	0.037
	medial collaterals	58/191 (30.4)	97/218 (44.5)	0.004
Nutcracker Phenomenon	Absent	120/157 (76.4)	189/200 (94.5)	0.000
	Anterior	14/157 (8.9)	4/200 (2)	
	Posterior	22/157 (14)	6/200 (3)	
	Combined	1/157 (0.6)	1/200 (0.5)	
Diameter of the ISV(mean) cm		4.00	3.96	0.378
Outflow angle of the ISV(mean)		103.66	108.30	0.076
Outflow angle of the ISV if Bährens type 2(mean)		110.00	106.46	0.885
Bähren classification**	Type 1	60/185 (32.4)	51/209 (24.4)	0.009
	Type 2	32/185 (17.3)	36/209 (17.2)	
	Type 2b	12/185 (6.5)	38/209 (18.2)	
	Type 3	20/185 (10.8)	12/209 (5.7)	
	Type 4a	11/185 (5.9)	18/209 (8.6)	
	Type 4b	45/185 (24.3)	44/209 (21.1)	
	Type 5	5/185 (2.7)	10/209 (4.8)	

* For some characteristics the number of patients does not equal the total number of adults or adolescents because we omitted patients in whom the characteristics could not be determined. ** The sum of type 2b and 4b is lower than the number of competent outflow valves, because the Bähren classification does not take into account insufficiencies that can only be proven by passing the competent outflow valve. Moreover 15 patients were excluded (nine adults and six adolescents) because their phlebography could not be classified according Bähren. Type 0 was encountered in six adults.

**Figure 2a:**
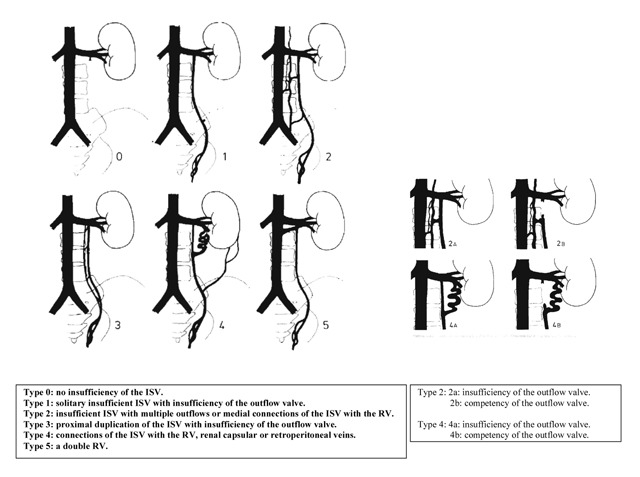
Bähren classification (left ISV) [Bahren, et al. 1992, *Rofo*, 157, 355–60] (with permission of Röfo, Thieme).

After analysis and comparison of about 500 phlebograms, we demonstrated that our suspicion was not unfounded. Adults had significantly more often a competent outflow valve than adolescents. Moreover, adult varicocele had a more complex anatomy consisting of a significantly higher number of collaterals and of a complex outflow in the renal vein. The phlebographic complexity of adult varicocele could be the remnant of an insufficient embryonic venous drainage during the ontogenesis of the left ISV.

Left-sided varicocele in many adolescents seems to be more a congenital abnormality rather than a progressive chronic disease: we found a higher rate of insufficient outflow valves, congenital renospermatic bypasses and the compressive nutcracker phenomenon.

On the other hand, we have arguments that right-sided varicocele might be a progressive disease. The incidence of an insufficient ISV increase with age (2.4 times more often in adults (33%) than in adolescents (14.7%). The diameter of the ISV also increases significantly with age: a fact that could point at a degenerative process with reflux and dilatation (Table 2b) (Figure 2b).

**Table 2b:** Phlebographic characteristics of the right insufficient ISV in adults and adolescents.

Phlebographic characteristics of the right ISV		Adolescents N = number adolescents/total N = 28 (%)*	Adults N = number adults/total N = 80(%)*	P-value

Spontaneous visualization of the ISV (IVC injection)		0/27 (0.0)	5/77 (6.5)	0.323
Spontaneous visualization of the ISV (RV injection)		4/27 (14.8)	11/78 (14.1)	1.000
Incompetence of the outflow valve (IVC injection)		1/26 (3.8)	5/77 (6.5)	1.000
Incompetence of the outflow valve (RV injection)		4/26 (15.4)	10/71 (14.1)	1.000
Reno-spermatic bypass	Absent	26/28 (92.9)	78/80 (97.5)	0.290
	Complete	2/28 (7.1)	2/80 (2.5)	
Mean nr. of competent valves below the outflow valve		0.11	0.23	0.514
Duplication of the ISV	Solitary ISV	12/28 (42.9)	37/80 (46.2)	0.304
	Multiple/duplication	16/28 (47.2)	43/80 (53.7)	
Paraspermatic veins		23/28 (82.1)	71/80 (88.8)	0.513
Collaterals	collaterals	13/28 (46.4)	40/80 (50)	0.744
	lateral collaterals	12/28 (42.9)	34/80 (42.5)	1.000
	medial collaterals	5/28 (17.9)	28/80 (35.4)	0.099
Outflow level of the ISV into the ICV	L1	0/28 (0)	3/79 (3.8)	0.964
	L1–L2	6/28 (21.4)	17/79 (21.5)	
	L2	10/28 (35.7)	30/79 (38)	
	L2–L3	9/28 (32.1)	20/79 (25.3)	
	L3	3/28 (10.7)	8/79 (10.1)	
	L3–L4	0/28 (0)	1/79 (1.3)	
Diameter of the ISV(mean) cm		3.51	3.99	0.023
Outflow angle of the ISV and the ICV (mean)°		31.04	25.13	0.070
Outflow angle of the ISV and RV (Siegel type 4a) (mean)°		110.60	90.83	0.383
Siegel classification	Type 1	9/28 (32.1)	29/79 (36.7)	0.532
	Type 2	8/28 (28.6)	21/79 (26.6)	
	Type 2a	6/28 (21.4)	13/79 (16.4)	
	Type 3	0/28 (0)	5/79 (6.3)	
	Type 4	1/28 (3.6)	7/79 (8.9)	
	Type 4a	4/28 (14.3)	4/79 (5.1)	

* For some characteristics the number of patients does not equal the total number of adults or adolescents because we omitted patients in whom the characteristics could not be determined.

**Figure 2b:**
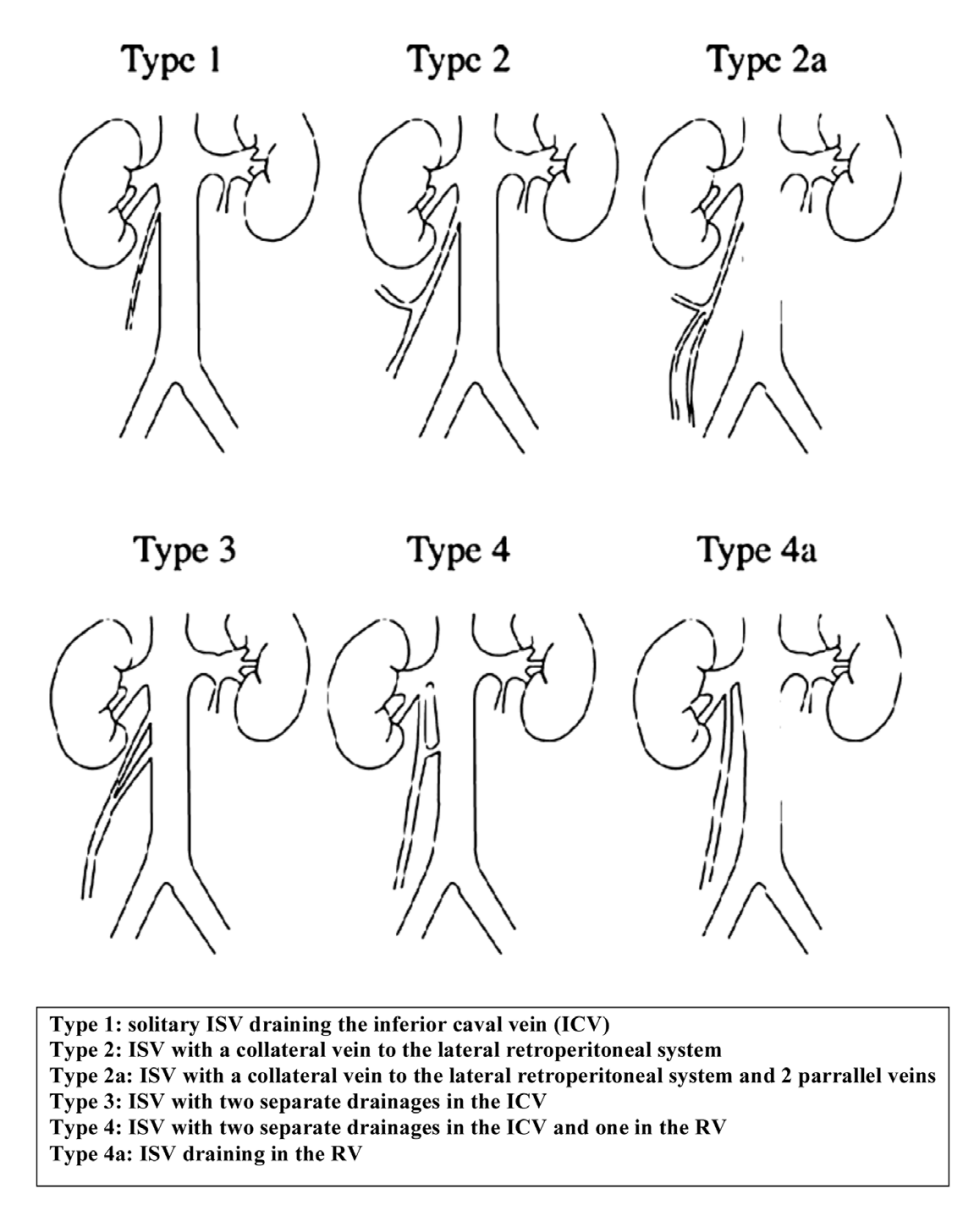
Siegel classification (right ISV) [Siegel, et al. 2006, *Cardiovasc Intervent Radiol*, 29, 192–7] (with permission of CVIR, Springer).

Many theories have been developed about how varicoceles can cause infertility. The causal association is most probably multifactorial, combining of lifestyle factors, genetic factors and the effect of reflux of blood into the PP.

The reflux of blood into the ISV is a direct result of the insufficiency of valves, a mechanism we can observe during the phlebography of varicocele patients. By reflux of warm blood, temperature in the testicle rises. Furthermore, in a varicocele patient standing upright, the hydrostatic pressure at the PP and the testicle increases according the height of the fluid column in the ISV. Venous drainage at the PP will then be impaired, and the cooling of the testicle less efficient. This hydrostatic *counterpressure* might interfere with the arterial and nutritional supply of the testicle and decrease spermatogenesis and testicle growth.

In an editorial of Andrologia, Gat titled that, “Erect posture of humans leads to infertilit.” [46]. In two other articles, he described that the increased pressure at the PP is only caused by the height of the blood column in the insufficient ISV. He calculated this hydrostatic pressure by the expected distance between PP and renal vein according the formula P = h × g × *ρ*, resulting in a value of 31.5 mm Hg at the left and a value of 27 mm Hg at the right side. This hydrostatic pressure is, according to Gat, high enough to cause testicular insufficiency and infertility in varicoceles. This is the theoretical point of view.

Pressure measurements in the PP or inguinal ISV of varicocele patients were previously performed by direct measurements through a needle or catheter in the PP [47, 48, 49]. The pressures that were measured in these studies were inconsistent and conflicting, in our opinion because of methodological errors. We aimed to measure pressures in the caudal ISV directly and to find out whether infertility can be explained by increased pressure.

**Figure 3:**
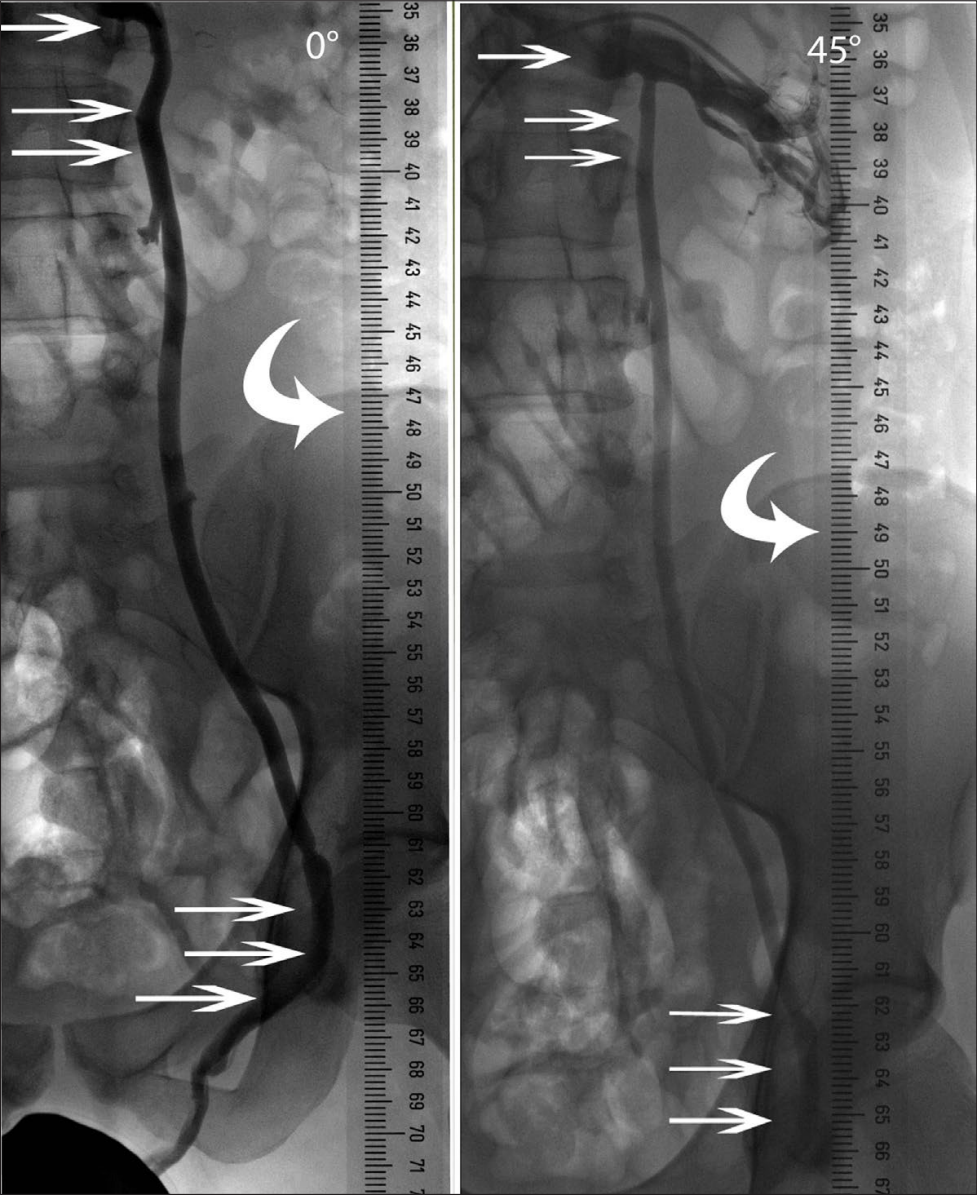
Locations of venous pressure measurements. Pressure measurements performed in the renal vein (small arrow), in the outflow (double small arrows) and inguinal segment (triple small arrows) of the internal spermatic vein in 0° and 45° position. Radio-opaque yardstick (curved arrow).

**Figure 4:**
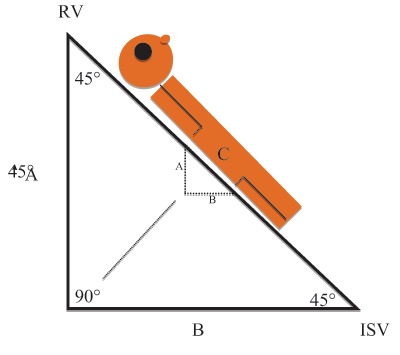
Extrapolation of 45° to 90°: (RV = renal vein/ISV = internal spermatic vein). The pressure P at 45° was calculated as *h = P /* ρ *× g* to obtain the height of the corresponding fluid column (A). This height (A) corresponds to the perpendicular side of a triangle, of which the oblique side (C) would correspond to the theoretical height in 90° erect position. This oblique side (C) can be calculated with the formula: *sin 45*° *= obtained height/oblique side*. Then the oblique side was put in the formula *P =* ρ *× g × h* to calculate the extrapolated pressure at 90° erect position.

Performing pressure measurements through a microcatheter in the ISV was not an easy task. Initial results were not at consistent. We tested and calibrated our method ex-vivo and standardized the measurement method. We compared pressures obtained via a microcatheter (external pressure transducer) with those registered with two different commercial pressure wires. In-vitro (in an experimental tube) and in-vivo (endovascular in the ISV) tests demonstrated that the direct measurements (pressure wires) could be reproduced by the microcatheter measurements (external pressure transducer) (Figure 3). Measurements were extrapolated to a 90° erect position (Figure 4)

For the first time, an in-vivo experiment done in 42 patients showed that the absolute mean pressure in the inguinal left ISV is 33 mm Hg. According to the hypothesis of Gat, this pressure would only exist of a hydrostatic pressure component. However, the height of the blood column that produces this hydrostatic pressure would reach far above the renal vein, even higher than the right atrium. We disclosed that the pressure in the renal vein and at the outflow of the ISV did not change with patient’s position. It was constant at about 12 mm Hg, corresponding to the systemic venous pressure. The absolute measured pressure is therefore composed of a hydrostatic (21 mm Hg), and a systemic pressure (12 mm Hg) component. This hydrostatic pressure was compatible with the indirect calculation from the measured height of the ISV (distance between inguinal ISV and renal vein).

As the normal capillary pressure of the testicle can be estimated at less than 20 mm Hg, then the hydrostatic pressure in upright standing position alone appears to be high enough to act as a counterpressure. This counterpressure could impair arterial supply and spermatogenesis in varicoceles.

Only standardized pressure measurements in fertile patients without varicoceles would be able to answer whether erect posture in varicocele patients leads to infertility.

## Conclusion and perspectives

The current commercially available tissue-adhesives, Histoacryl transparent and Glubran2, are equally efficient, safe and tolerable for transcatheter embolization of varicoceles. Glue can cause a mild, self-limiting pain in 1/3 of the patients in the week after embolization, independently of what product was used. Because Glubran2 has a CE label for intravascular use, it is medico-legal justified to use this glue. Worldwide, glue is not a popular embolic agent for the treatment of varicoceles, probably because it requires more skills or preconception persists about the potential complications. Controlled randomized trials, reporting the success and complications rate, as well as the pregnancy rate could lead to an FDA approval for intravascular use and contribute to the attractiveness of glue.

Onyx has the potential of an efficient and safe agent for the embolization of varicoceles. Unfortunately, the acute pain reaction as well as the radiation exposure during embolization have to find a solution before Onyx can be recommended. Finally, we have to explore in which anatomical situation Onyx can constitute an advantage over the other embolics.

Radiation exposure is safe during embolizations of varicoceles with highly viscous liquid products. We report a gonadal dose that is far below the lower threshold corresponding to deterministic effects. In future, by the use of the flat panel detectors, the radiation exposure may be reduced even further.

In left-sided varicocele children’s spermatic vein showed more often valve insufficiency, renospermatic bypasses and nutcracker syndrome. In adults, reflux more often is caused by collateral connections. Left varicocele seems rather a congenital abnormality than an evolutionary disease as commonly believed. At the right varicocele, we have shown that the diameter of the ISV increases in adults and that a bilateral varicocele occurs 2.4 times more. As far as the right varicocele is concerned, we do have arguments for an evolutionary chronic disease. In a subsequent study, we could further investigate and discover links which might predict whether certain children may profit from a prophylactic embolization.

This thesis demonstrated in-vivo that the mean absolute pressure in the inguinal ISV in the upright position is significantly high enough to build a counter pressure to the capillary pressure, and hence impair the spermatogenesis. In addition, we have invalidated Gat’s hypothesis and proven that not only the hydrostatic pressure is responsible for this elevated pressure, but the sum of the systemic and the hydrostatic pressure. We could investigate whether clinical presentation, thermography, CDUS, phlebographic anatomy or age are associated with different pressure levels. Here again, the detection of certain sub-groups could change the therapy policy.

However, we still ignore whether the pressures we obtained, are *really* pathological since we cannot compare with a healthy control population. We could measure the pressure in the ISV of a control group of fertile men without varicocele. It will not be an easy task to convince the ethics committee. Moreover, it will be a technical challenge to catheterize healthy men retrograde through several competent valves down to the inguinal ISV. Only then we could answer the question “does erect posture in varicoceles lead to infertility?”
